# Blockchain-Enabled Self-Sovereign Identity Applications in Health Care: Scoping Review

**DOI:** 10.2196/89574

**Published:** 2026-05-26

**Authors:** Abha Pokharel, Surya Kathayat

**Affiliations:** 1 Norwegian University of Science and Technology Trondheim, Trøndelag Norway

**Keywords:** blockchain, decentralization, health care, identity management, privacy, security, self-sovereign identity

## Abstract

**Background:**

Self-sovereign identity (SSI) provides a decentralized approach to digital identity management, enabling individuals to control their personal data without reliance on centralized authorities. Blockchain technology offers a tamper-resistant and distributed infrastructure that can support secure and verifiable identity systems. In health care, where identity fragmentation, privacy risks, and interoperability challenges persist, blockchain-enabled SSI (BC-SSI) has been proposed as a potential solution. However, existing research remains heterogeneous, with varying levels of technical maturity and limited evidence of real-world deployment.

**Objective:**

This study conducts a scoping review to systematically map BC-SSI applications in health care and to analyze their application domains, development stages, study aims, targeted challenges, and technological infrastructures. In addition, this study aims to identify structural gaps in current research and assess the readiness of BC-SSI systems for clinical deployment.

**Methods:**

This review followed the PRISMA-ScR (Preferred Reporting Items for Systematic Reviews and Meta-Analyses Extension for Scoping Reviews) methodology. A comprehensive literature search conducted between September 2024 and August 2025 identified 37 peer-reviewed studies that met predefined inclusion criteria. Data were extracted and synthesized using descriptive and thematic analyses across application areas, system maturity, technological components, and reported challenges.

**Results:**

The findings indicate that BC-SSI research in health care remains at an early stage of maturity, with most studies proposing conceptual models or prototype implementations and limited real-world validation. Applications predominantly focus on identity verification, credential management, and privacy-preserving data exchange across domains such as electronic health records, mobile health, and access control systems. Commonly used technologies include decentralized identifiers, verifiable credentials, smart contracts, and privacy-enhancing mechanisms such as zero-knowledge proofs and selective disclosure. Despite rapid technical development, persistent challenges include interoperability limitations, governance gaps, usability concerns, and insufficient integration with health care infrastructures. Notably, a structural gap was identified between technological capability and system-level readiness for clinical deployment.

**Conclusions:**

BC-SSI technologies demonstrate potential for enabling secure, interoperable, and patient-centric identity management in health care. However, current research is predominantly technology-driven and lacks sufficient system-level validation. This study highlights the need for integrated architectural approaches, governance frameworks, and real-world evaluation to bridge the gap between conceptual innovation and clinical implementation. Advancing BC-SSI toward health care adoption will require coordinated progress across technical, organizational, and regulatory dimensions.

## Introduction

### Overview

Blockchain-enabled self-sovereign identity (SSI) applications in health care are attracting greater research attention as health data becomes increasingly digital and the need for secure, patient-focused identity management grows. Early blockchain projects in health care primarily focused on maintaining record immutability and enabling secure data exchange. More recently, researchers have explored the use of SSI to give patients greater control over their digital identities and health information [1-3]. The move from centralized to decentralized identity systems is driven by ongoing problems in current health care systems, such as limited interoperability, privacy risks, and fragmented access control [4-6]. These issues are especially evident in situations such as referrals, emergency care, and telehealth, where rapid and reliable identity verification is needed for effective clinical coordination [7].

The need for stronger digital identity systems is clear from the rise in health care data breaches. Growing regulatory demands, such as those under the General Data Protection Regulation (GDPR) and the Health Insurance Portability and Accountability Act (HIPAA), also underscore the need for better solutions [1,8,9]. In this context, BC-SSI systems ensure this by enabling decentralized identity checks, secure credential management, and private data sharing among health care providers. Still, there are trade-offs between decentralization, scalability, governance, and system performance. These challenges are even more complex in regulated health care settings [10,11].

Even though more research is being conducted on BC-SSI in health care, the studies remain scattered. Most studies focus on concepts or early prototypes [1,12,13]. Few studies bring together architectural designs, technology choices, platforms, and governance to assess whether these systems are ready for real-world health care use. However, these studies have rarely examined how BC-SSI fits with clinical systems, regulations, and daily operations. As a result, there is an incomplete understanding of how practical these systems are in real-world health care settings. To fill this gap, this study makes 4 main contributions. First, it reviews peer-reviewed literature to map out BC-SSI applications in health care. Second, it looks at their technology, development stages, and use. Third, it brings together common architectural features. Fourth, it creates a layered architectural model and assesses the readiness of current BC-SSI systems for real-world health care and clinical use.

### Background

#### SSI and Blockchain

SSI is a decentralized identity model that enables individuals to create, manage, and selectively disclose digital credentials without reliance on centralized identity providers [7,13]. In SSI systems, identity attributes are not stored within institutional databases but are instead held by individuals as verifiable credentials (VCs), which can be presented when required. These systems operate within a trust framework involving issuers (eg, health care institutions), holders (eg, patients), and verifiers (eg, service providers), supported by governance mechanisms such as credential standards, trust registries, and revocation processes. In health care environments, traditional identity systems remain largely centralized and fragmented, requiring repeated identity verification across multiple organizations. This results in inefficiencies, data duplication, and increased risks of identity mismatch and unauthorized access, increasing vulnerability to data breaches. These limitations have driven interest in decentralized identity models such as SSI for health care applications.

Blockchain technology provides the underlying infrastructure that supports SSI through immutable and verifiable record keeping [10]. Within BC-SSI architectures, decentralized identifiers (DIDs) represent user-controlled identities, while VCs enable cryptographic validation of identity attributes without direct reliance on issuing authorities. Blockchain is typically used to anchor DID registries, credential schemas, and revocation mechanisms, while sensitive health data remains off chain to preserve privacy. This combination enables secure and tamper-evident identity verification across distributed health care systems. To operationalize these capabilities, BC-SSI architectures incorporate a set of supporting mechanisms. These include selective disclosure for controlled data sharing, zero-knowledge proofs (ZKPs) for privacy-preserving verification, and smart contracts for automated enforcement of access control and audit policies. Together, these mechanisms enable decentralized identity management while addressing key requirements of trust, security, and accountability in health care contexts.

In studies of health care identity systems, security and privacy are closely related yet distinct concerns. Security focuses on ensuring confidentiality, integrity, and availability of identity and health data through mechanisms such as authentication and access control. Privacy emphasizes minimizing unnecessary disclosure of sensitive information and enabling individuals to control how their data are shared [11,12]. These concerns are particularly critical in health care due to the sensitivity of medical data and the complexity of cross-organizational data exchange [1].

#### SSI Frameworks and Blockchain Network Types

SSI frameworks define the architectural components required for credential issuance, storage, verification, and trust management. Implementations commonly use platforms such as Hyperledger Indy and Aries to support decentralized identity registries and secure agent-based communication [7,12]. SSI architectures may operate on different blockchain network configurations depending on governance and trust requirements. Public (permissionless) networks such as Bitcoin and Ethereum enable unrestricted participation and transparent validation. Consortium networks restrict participation to authorized entities and are often deployed in multi-institutional collaborations. Private networks are centrally governed and typically implemented within a single organization. These models reflect varying trade-offs between decentralization, scalability, and governance control relevant to identity system design.

#### Smart Contracts

Smart contracts are programmable components deployed on blockchain platforms that automate identity-related operations. In health care contexts, they can enforce access control policies, manage consent conditions, and record auditable transaction logs without manual intervention [13]. This supports transparent and accountable identity management across distributed health care systems.

Despite increasing research activity in BC-SSI, existing studies remain heterogeneous in terms of application focus, architectural design, and implementation maturity. Many proposed solutions are limited to conceptual models or prototype implementations, with minimal evaluation in real-world health care environments. Furthermore, there is limited consolidated analysis of how BC-SSI mechanisms address health care-specific challenges such as interoperability, governance, and clinical workflow integration.

To address this gap, this study conducts a scoping review to systematically map BC-SSI applications in health care, focusing on application domains, technological components, development stages, and the challenges these systems aim to address.

## Methods

### Study Design

We used the scoping review methodology to map existing proposals that integrate BC-SSI into health care. A scoping review is particularly appropriate because BC-SSI research is still an emerging area, conceptually diverse, and lacking consolidated empirical evidence. The review followed the framework of Arksey and O’Malley [14], as enhanced by Levac et al [15], and adhered to the PRISMA-ScR (Preferred Reporting Items for Systematic Reviews and Meta-Analyses Extension for Scoping Reviews) reporting guidelines [16]. The completed PRISMA-ScR checklist is provided in Multimedia Appendix 1. The methodology includes five stages: (1) defining research questions (RQs), (2) identifying relevant studies, (3) selecting eligible publications, (4) charting extracted data, and (5) synthesizing and reporting findings. The following 5 RQs guided the review:

- RQ1: What BC-SSI application areas and data types are addressed in health care?
- RQ2: What development stages (proposal, architecture, and prototype) are used in BC-SSI studies?
- RQ3: What are the main aims of BC-SSI applications in health care?
- RQ4: What challenges do BC-SSI technologies aim to address?
- RQ5: What BC-SSI technologies, frameworks, and infrastructures are used?

### Search Strategy and Study Selection

A comprehensive search was conducted across 6 databases, along with their distribution as illustrated in Table 1. Google Scholar was included to capture interdisciplinary publications that may not be indexed in domain-specific databases. The final search update was performed on August 15, 2025. Search strings combined free-text terms related to “self-sovereign identity,” “blockchain,” and “healthcare” using Boolean operators (“OR” within concept groups and “AND” between concept groups). Synonym groups (eg, health, eHealth, medical, hospital, and clinical) were expanded accordingly. Backward and forward snowballing were conducted to identify additional relevant studies [17]. Full search strings are provided in Multimedia Appendix 2. A total of 345 records were initially retrieved. The distribution of retrieved records by database is presented in Table 1.

**Table 1:** Captured works of literature by source (N=345).

Source	Captured works, n
Scopus	95
Web of Science	46
Google Scholar	133
Embase	19
Medline	13
IEEE Xplore	39

### Eligibility Criteria and Study Selection

Peer-reviewed studies published between 2015 and 2025 were considered eligible. Inclusion criteria included BC-SSI proposals addressing health domain problems (conceptual model, framework, proposal, prototype, experiment, implementation, and pilot), technical descriptions (eg, architecture, prototype, and framework), English-language peer-reviewed primary studies, and studies published between 2015 and 2025. Exclusion criteria included studies unrelated to health care, blockchain-only solutions without SSI components, nonprimary studies (eg, reviews and editorials) or abstract-only publications, and non-English publications.

All references were imported into Zotero (Corporation for Digital Scholarship) for deduplication and screened using Rayyan [18]. Two reviewers independently screened titles, abstracts, and full texts according to predefined criteria. Disagreements were resolved through discussion and consensus to ensure consistent interpretation.

After duplicate removal and eligibility screening, 33 studies were retained. A total of 4 additional studies were identified through snowballing, resulting in 37 studies included in the final synthesis.

### Data Extraction and Charting

Data were charted using a predefined extraction matrix consistent with PRISMA-ScR guidance. The first author performed data extraction, and the second author reviewed all entries. Discrepancies were resolved through discussion, and all final decisions were agreed jointly. The following data were extracted: general information, including author(s) ID and study; publication type, including journal, conference, proceedings, or book chapter; application area (eg, health records, mobile health [mHealth], wearable/embedded systems, health care services); data types handled (eg, medical data, credentials, vaccination records, and location data); and development stage, including proposal, architectural design, and prototype/experimental implementation.

Extracted variables were mapped directly to the RQs: application areas and data types informed RQ1; development-stage classification addressed RQ2; thematic coding of study aims addressed RQ3; reported challenges informed RQ4; and BC-SSI–enabling technologies, blockchain platforms supporting SSI, blockchain types, smart contracts, and storage models were analyzed under RQ5.

Following Petersen et al [19], classification categories for application areas, data types, and development stages were iteratively developed and refined during the charting process. Categories were merged or adjusted as necessary to ensure conceptual consistency and reduce overlap across studies.

### Data Synthesis and Reporting

After screening and data extraction, findings were synthesized descriptively and thematically, aligned with the 5 RQs (RQ1-RQ5). Descriptive statistics were used to summarize distributions across application areas, data types, development stages, and technological components. Thematic categorization was applied to study aims and reported challenges to identify recurring patterns across BC-SSI implementations. The complete list of included studies and their extracted characteristics is presented in the Results section.

## Results

### Overview of Included Studies

**Figure 1:**
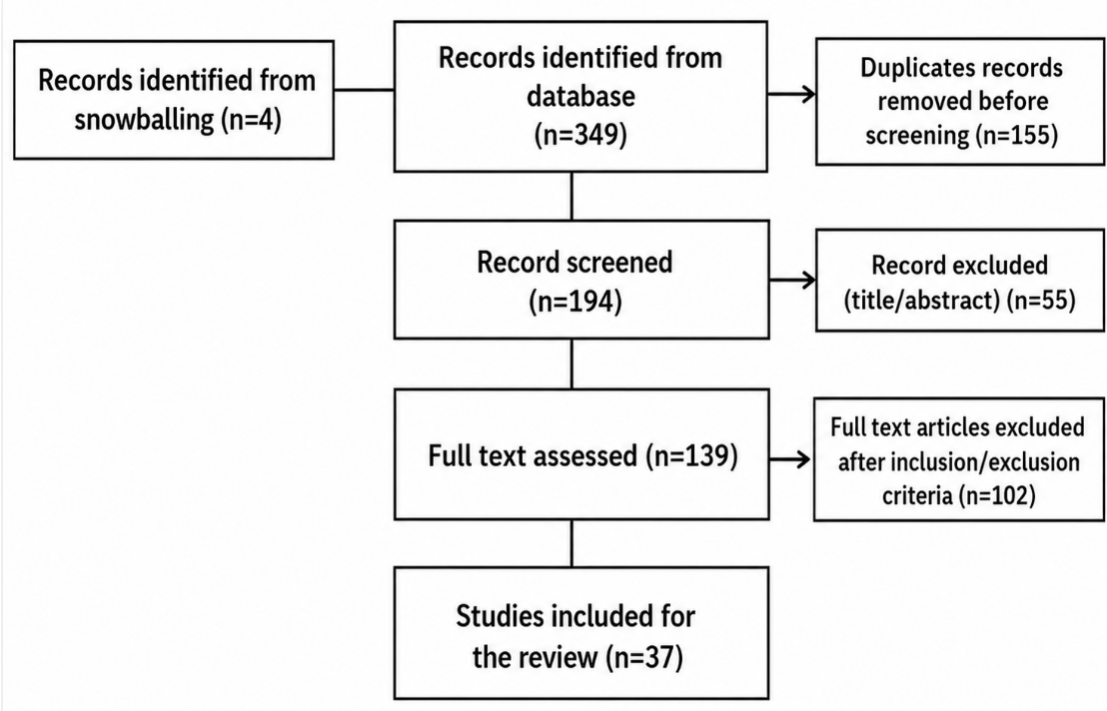
PRISMA-ScR (Preferred Reporting Items for Systematic Reviews and Meta-Analyses) flow diagram of study selection.

A total of 37 studies met the inclusion criteria and were included in the final synthesis. Figure 1 presents the PRISMA-ScR flow diagram summarizing the study selection process. Table 2 presents the key characteristics of the included studies, including publication type, application area, data types addressed, and development stage.

**Table 2:** Included research papers in the scoping review and their key characteristics.

ID	Study (type)	Application area	Data types	Development stage
1	Nasrin [20] (conference)	Health records	Vaccination and credential	Proposal
2	Hak et al [21] (conference)	Embedded and wearable systems	Sensitive and credential	Architecture
3	Fotopoulos et al [22] (conference)	Embedded and wearable systems	Credential	Proposal
4	Zou et al [23] (journal)	Embedded and wearable systems	Credential	Prototype/experimental
5	Bai et al [24] (journal)	Embedded and wearable systems	Medical and credential	Prototype/experimental
6	Bandara et al [25] (conference)	mHealth^a^	Location and sensitive	Prototype/experimental
7	Kormiltsyn et al [26] (conference)	Health services	Medical	Architecture
8	Harrell et al [12] (journal)	mHealth	Sensitive	Architecture
9	George and Chacko [27] (journal)	mHealth	Sensitive and credential	Prototype/experimental
10	Sahi et al [28] (conference)	Health records	Credential and sensitive	Proposal
11	Kim et al [29] (conference)	mHealth	Credential	Architecture
12	Abid et al [30] (journal)	mHealth	Sensitive	Prototype/experimental
13	Popa et al [31] (journal)	Embedded and wearable systems	Sensitive and credential	Prototype/experimental
14	Saha et al [32] (conference)	Health records	Credential	Proposal
15	Rafid et al [33] (conference)	mHealth	Credential and medical	Proposal
16	Saragih et al [4] (conference)	Health records	Sensitive and credential	Proposal
17	Santos et al [9] (journal)	Health records	Medical and credential	Prototype/experimental
18	Song et al [34] (journal)	Embedded and wearable systems	Sensitive and credential	Prototype/experimental
19	Keil et al [35] (conference)	Embedded and wearable systems	Credential	Architecture
20	Freytsis et al [36] (journal)	Access control	Credential	Architecture
21	Zhuang et al [37] (journal)	mHealth	Sensitive and credential	Architecture
22	Al Muharif and Ahmed [38] (conference)	Health records	Medical and credential	Prototype/experimental
23	Pujari et al [39] (conference)	Health records	Credential and sensitive	Architecture
24	Tcholakian et al [8] (journal)	Health records	Medical	Architecture
25	Sami et al [6] (conference)	Health records	Medical	Proposal
26	Wang et al [40] (journal)	Health services	Credential	Prototype/experimental
27	Kang et al [41] (journal)	Health services	Location and credential	Architecture
28	Pujari et al [42] (journal)	Clinical research	Credential	Prototype/experimental
29	Manoj et al [43] (journal)	Health services	Credential	Prototype/experimental
30	Abubakar et al [44] (conference)	Health records	Credential	Prototype/experimental
31	Mchale et al [45] (conference)	Health records	Vaccination	Prototype/experimental
32	Guerar et al [46] (journal)	Health records	Credential and medical	Proposal
33	de Oliveira et al [47] (conference)	mHealth	Credential and medical	Prototype/experimental
34	Thirasak et al [48] (journal)	Health records	Medical	Prototype/experimental
35	Makina et al [1] (conference)	Health records	Medical	Architecture
36	Boi et al [49] (journal)	Health records	Credential and medical	Prototype/experimental
37	Saidi et al [50] (journal)	Health records	Medical	Prototype/experimental

^a^mHealth: mobile health.

### Descriptive Characteristics of Included Studies

Across the 37 included studies, 49% (18/37) were conference publications and 51% (19/37) were journal articles. Google Scholar contributed the largest share of the initially retrieved records (133/345, 39%), whereas Embase contributed the smallest share (19/345, 6%). Among the final set of included publications, Scopus accounted for 65% (24/37) and IEEE Xplore for 19% (7/37). Publication activity peaked in 2022 for both conference papers (6/18, 33%) and journal papers (5/19, 26%), reflecting growing interest in BC-SSI solutions in health care.

### RQ1: BC-SSI Application Areas and Data Types

The review identified 6 major BC-SSI application areas in health care. Health records constituted the largest category (16/37, 43%), encompassing applications related to personal health data management, medical record exchange, and credential verification. mHealth applications represented the second-largest category (8/37, 22%), reflecting the growing use of decentralized identity mechanisms in mHealth platforms and patient-facing applications.

Embedded and wearable systems accounted for 19% (7/37) of the studies and included applications involving the Internet of Medical Things and smart medical devices. These systems often require secure device authentication and identity verification in distributed health care environments. The remaining application areas included health care services (4/37, 11%), clinical studies (1/37, 3%), and facility access control (1/37, 3%).

The studies also addressed several types of health care–related data. Credential data were the most frequent type (27/37, 73%), highlighting the central role of identity verification and authorization in BC-SSI architectures. Medical and other sensitive personal data were commonly associated with health record management and wearable device applications. Location data appeared primarily in mHealth and contact-tracing scenarios, in which decentralized identity mechanisms were used to support privacy-preserving monitoring and verification.

### RQ2: Development Stages of BC-SSI Applications

The reviewed studies demonstrate varying levels of development maturity in BC-SSI health care applications. Across 37 studies, 22% (8/37) presented conceptual proposals, 30% (11/37) provided architectural designs, and 49% (18/37) provided prototype or experimental implementations. Although more than one-half of the studies reported prototype-level implementations, most evaluations were limited to simulated environments or small-scale user testing, indicating early-stage technological validation. Only a small number reported real-world deployment or evaluation.

### RQ3: Aims of BC-SSI Studies

Thematic analysis of the reviewed studies indicates that BC-SSI research is primarily driven by 3 major objectives. Privacy preservation was the most frequently reported aim, accounting for approximately 38% (14/37) of the studies, followed by secure data management (7/37, 19%) and patient-centric identity control (7/37, 19%). The distribution of these aims is summarized in Table 3.

**Table 3:** Mapping of included studies to research objectives (research question 3 [RQ3]) and corresponding health care challenges (research question 4 [RQ4]).

ID	Study aim (RQ3)	Challenges (RQ4)
1	Vaccination verification and authentication	I^a^, DI^b^, DP^c^, S^d^
2	Anonymous cross-domain authentication	I, P^e^, E^f^, DI, DP (IoMT^g^)
3	Device authentication and revocation (IoMT)	DI, I, SC^h^, E, S
4	Privacy-aware authentication and data exchange	I, DI, S, SC, E
5	Decentralized access control for health data	AC^i^, SC, E, P, A^j^
6	Privacy-aware contact tracing	P, SC, I, T^k^, DP
7	Secure authentication for medical data exchange	DM^l^, AC, S
8	Patient-centric data management and sharing	IDM^m^, I, AC, A, DP
9	Credential issuance and access control	AC, I, A, P, S, DP
10	Consent-based access control	IDM, CM^n^, I, E, P, S
11	Identity tracking and transparency	IDM, T, DP, A
12	Privacy-preserving certificate verification	DI, DP, A, P
13	Consent-based identity sharing	IDM, I, A, P, S
14	Decentralized credential verification	IDM, P, S, E, DP, A
15	Identity management (rare diseases)	IDM, S, E
16	Cross-institution authentication	DM, S, P, I
17	Identity verification for service access	DM, S, I, P
18	Identity management for users and devices	IDM, A, P, S, DI
19	Attribute-based credential verification	DM, A, P, S, I
20	Anti-profiling access control	A, AC, P
21	Identity provisioning for newborns	P, S
22	Patient-controlled identity and data	P, S, I, A, E
23	Patient-centric credential preservation	DM, P, A
24	Secure EHR^o^ exchange	DI, P, S, A
25	Fine-grained access control	AC, P, S, E
26	Credential validation and access control	IDM, P, S, A
27	Unlinkable contact tracing	P, A, E
28	Identity verification for research access	AC, IDM, I, DP, P
29	Secure identity validation	IDM, DP, S, P, A
30	Identity management and authentication	S, P, CM
31	Fraud-resistant certificate verification	S, T, DP, A, DM
32	Identity recovery mechanisms	IDM, S, A
33	Patient-centric data preservation	IDM, P, I, A
34	Decentralized data access control	P, S, AC, I
35	Transparent access control	P, S, T
36	Scalable EHR access control	S, E, SC
37	Privacy-aware access control	AC, P, S, A

^a^I: interoperability.

^b^DI: data integrity.

^c^DP: data provenance.

^d^S: security.

^e^P: privacy.

^f^E: efficiency.

^g^IoMT: Internet of Medical Things.

^h^SC: scalability.

^i^AC: access control.

^j^A: autonomy.

^k^T: transparency.

^l^DM: data management.

^m^IDM: identity management.

^n^CM: consent management.

^o^EHR: electronic health record.

Privacy-related aims focus on enabling controlled disclosure of identity and health-related attributes during verification and data exchange processes. Secure data management aims emphasize strengthening authentication, credential validation, and integrity assurance within health care systems. Patient-centric identity control aims are centered on enabling individuals to manage their digital identities, credentials, and consent in interactions with health care providers.

Less frequently, studies addressed objectives such as secure electronic health record (EHR) exchange, prevention of identity traceability, rare-disease identity management, and research identity verification. Overall, these findings indicate that BC-SSI research is primarily oriented toward enhancing privacy and identity assurance, with comparatively less emphasis on system-level integration and interoperability.

To explicitly link the study aim (RQ3) to the underlying health care challenges (RQ4), Table 3 provides a structured mapping of each included study to its primary aims and the corresponding challenges it addressed.

### RQ4: Privacy and Security Challenges Addressed by BC-SSI

Privacy (27/37, 73%) and security (26/37, 70%) were the most frequently addressed challenges across the reviewed studies. These reflect persistent limitations in traditional health care identity and data management systems.

Privacy-related challenges primarily involve overdisclosure of sensitive patient information, limited user control over data sharing, and the risk of identity linkability across health care providers. For example, vaccination and health certification systems [30,45] address the need for attribute-level verification without exposing full identity records through selective disclosure and the use of VCs. Similarly, decentralized access control frameworks [39,40,50] emphasize patient-controlled identity and consent management, while contact tracing and IoMT scenarios [25,41] focus on reducing identity traceability using pseudonymous identifiers and privacy-preserving verification mechanisms.

Security-related challenges center on weak authentication, unauthorized data access, and risks of credential forgery and tampering in distributed health care environments. Cross-domain authentication schemes [21-23] address identity verification weaknesses across health care systems, whereas blockchain-based access control and audit mechanisms [48,50] enhance accountability and authorization. In addition, vaccination systems and fraud-resilient health care platforms [30,45,47] mitigate credential forgery through VCs and immutable blockchain registries.

Overall, the findings indicate that BC-SSI research is primarily driven by confidentiality, integrity, and identity assurance requirements, whereas challenges related to scalability and operational performance remain comparatively less explored.

### RQ5: BC-SSI Technologies and Infrastructures

#### DIDs, VCs, and Digital Wallets

**Figure 2:**
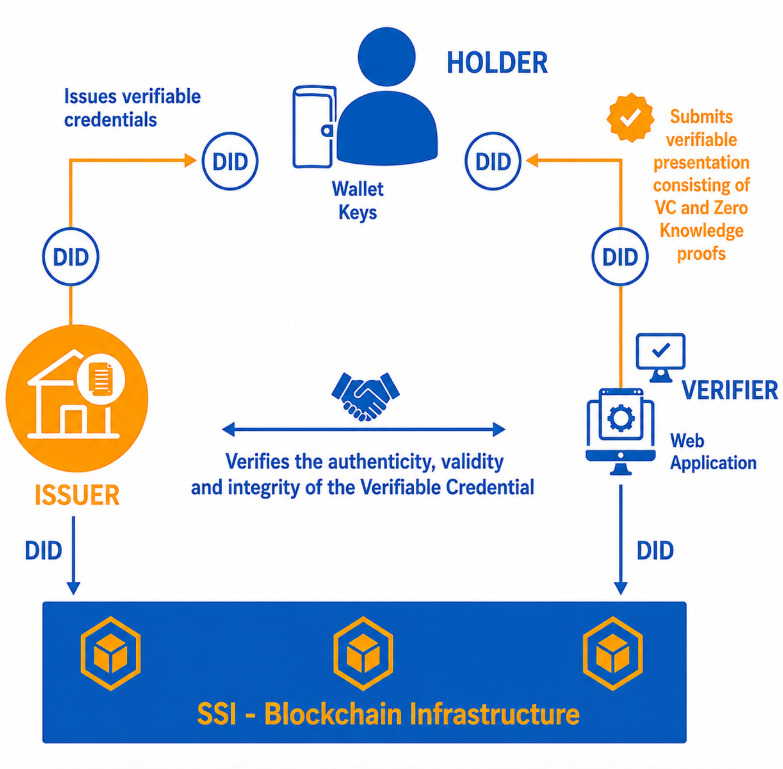
Blockchain-Enabled Self-Sovereign Identity (BC-SSI) technologies applied in health care. DID: decentralized identifier; VC: verifiable credential.

Across the reviewed literature, DIDs serve as identity anchors for patients, providers, and devices, enabling portable, cryptographically verifiable claims across institutional boundaries, as illustrated in Figure 2. VCs encode health care attributes, including vaccination status, professional qualifications, and access permissions. Selective disclosure mechanisms were widely used to support privacy-preserving verification.

Digital wallets were used to manage keys, credentials, and DID-based connections. Both custodial (server-managed) and noncustodial (client-side) models were observed, reflecting trade-offs between usability and security control.

#### Blockchain Platforms Supporting SSI

As illustrated in Table 4 , Hyperledger Indy, often combined with Hyperledger Aries, was the most frequently reported SSI-supporting platform (10/37, 27%). Hyperledger Fabric (8/37, 22%) was commonly selected for permissioned consortium settings. Ethereum-based solutions (6/37, 16%) leveraged smart-contract programmability but raised privacy and cost concerns. Notably, 32% (12/37) of studies did not explicitly specify the BC platform, limiting reproducibility and comparative assessment.

**Table 4:** Blockchain platforms and characteristics of blockchain-enabled self-sovereign identity (BC-SSI) implementations.

BC platform	Studies	Key characteristics
Hyperledger Indy (often with Hyperledger Aries)	[3], [5], [8], [9], [21], [25], [28], [29], [30]	Self-sovereign identity–oriented identity management, decentralized identifiers, verifiable credentials, and permissioned identity ecosystems
Hyperledger Fabric	[1], [49], [10], [11], [13], [15], [29]	Permissioned consortium blockchain, modular architecture, enterprise access control, and privacy-preserving transactions
Hyperledger Besu	[16]	Ethereum-compatible permissioned blockchain supporting enterprise deployment
Ethereum blockchain	[7], [8], [10], [19], [31]	Smart contract programmability, decentralized application support, and public blockchain infrastructure
Not specified (N/A)	[14], [17], [18], [20], [22], [23], [50], [26], [27], [2], [32]	Platform details not explicitly reported

#### Blockchain Types, Smart Contracts, and Storage Models

Private or permissioned blockchains were used in approximately 51% (19/37) of the reviewed studies. Public blockchain and consortium models were reported less frequently, whereas 43% (16/37) of studies did not explicitly specify the blockchain type. Smart contracts were implemented in 43% (16/37) of studies, primarily for consent management, credential issuance, revocation, and access control.

To reduce on-chain exposure of sensitive health data, 16% (6/37) of studies adopted a hybrid storage architecture. In these approaches, blockchain was used to store identifiers, hashes, or audit logs, whereas medical records were maintained off chain using distributed storage systems such as Interplanetary File System or institutional databases. Privacy-enhancing cryptographic techniques were reported in 35% (13/37) of studies, including ZKPs and selective-disclosure mechanisms that support attribute verification without revealing full data sets.

### Comparative Evaluation of BC-SSI Platforms

Table 5 summarizes the comparative characteristics of BC-SSI platforms identified in the reviewed studies. Hyperledger Indy was most frequently used in identity-centric implementations and provides native support for DIDs and VCs. Hyperledger Fabric was commonly selected in permissioned consortium settings but does not natively implement World Wide Web Consortium DID/VC standards. Ethereum-based solutions provide programmability for smart contracts and public-chain deployment models. Hyperledger Besu supports both permissioned and public configurations and is Ethereum compatible. uPort, built on Ethereum, provides decentralized identity functionality through wallet-based implementations. A notable proportion of studies did not explicitly specify the underlying blockchain platform or SSI framework.

**Table 5:** Comparative evaluation of blockchain-enabled self-sovereign identity (BC-SSI) platforms used in the literature.

Framework	DID^a^	VC^b^	Chain	Smart contract	Health care suitability	Key limitations
Hyperledger Indy	Strong	Strong	Permissioned	Limited	High (privacy-first and identity-centric)	No general-purpose smart contracts
Hyperledger Fabric	Indirect	Limited	Permissioned	Yes	High (enterprise-grade and consortium use)	No native DID/VC support
Ethereum	Strong	Strong	Public	Yes	Medium (high programmability)	Gas fees and public ledger visibility
Hyperledger Besu	Strong	Strong	Permissioned or public	Yes	Medium-high	Deployment and performance complexity
uPort	Strong	Strong	Public Ethereum	Yes	Medium	Dependent on Ethereum scalability
Not specified or custom models	Varies	Varies	Mixed	Varies	Not assessable	Low reproducibility and unclear stack

^a^DID: decentralized identifier.

^b^VC: verifiable credential.

Taken together, the technologies, infrastructures, and governance elements identified across the reviewed studies suggest that BC-SSI health care systems consist of several interacting technical and operational components. These recurring elements are synthesized into a conceptual layered architecture for BC-SSI deployment in health care contexts.

## Discussion

### Reflection on Principal Findings

This scoping review identified 37 peer-reviewed studies investigating BC-SSI applications in health care, revealing a rapidly growing yet structurally fragmented research landscape and indicating uneven development across technical and operational dimensions. Although publication activity has increased substantially since 2020, most contributions remain conceptual models, architectural proposals, or prototype implementations evaluated in controlled environments. Large-scale deployments, cross-institutional pilots, and longitudinal evaluations remain rare. This demonstrates that current BC-SSI research remains largely disconnected from real-world health care implementation. This review addresses the identified gap by providing a system-level synthesis of BC-SSI architectures and their readiness for health care deployment.

Across the reviewed literature, BC-SSI systems primarily focus on identity verification, credential management, consent-based access control, and privacy-preserving data exchange. These objectives correspond to longstanding challenges in health care identity management, including fragmented identity infrastructures and centralized trust models. However, a clear translational gap emerges between theoretical benefits and practical deployment, indicating that the proposed solutions have not yet been validated in operational health care settings. While many studies emphasize improved privacy, autonomy, and secure credential exchange, empirical evidence demonstrating integration with health care workflows, regulatory compliance, and cross-organizational interoperability remains limited, indicating insufficient evidence for real-world applicability and system-level integration.

Importantly, technical feasibility does not necessarily translate into operational readiness. Although several studies demonstrate working credential issuance and verification workflows, most systems have not evaluated within real clinical environments or integrated with EHR infrastructures. As a result, the readiness of BC-SSI systems for operational health care deployment remains uncertain, highlighting a lack of clinical validation and feasibility.

### Security and Privacy Mechanism in Operational Context

A recurring theme in the reviewed studies is the use of cryptographic mechanisms, including DIDs, VCs, selective disclosure, ZKPs, and smart contracts, to enhance privacy and security. However, these mechanisms are often discussed in the abstract without sufficiently examining their operational implications for health care systems, limiting understanding of their behavior in real-world health care settings. In a real health care setting, selective disclosure enables patients to demonstrate specific attributes, such as vaccination status and professional authorization, without revealing full identity records. ZKPs can support verification while minimizing unnecessary data exposure, potentially reducing the risk of large-scale data breaches. Smart contracts may automate consent enforcement and audit logging, theoretically enhancing traceability and accountability. Yet, these technical advantages introduce new risk vectors, demonstrating that enhanced privacy mechanisms may also increase system complexity and operational risk. Wallet compromise, key loss, credential revocation complexity, cross-institutional correlation attacks, and emergency override requirements represent unresolved operational challenges. Few studies systematically evaluate how these mechanisms perform under health care-specific constraints such as high patient turnover, emergency access demands, and strict compliance frameworks. Notably, to our knowledge, no studies have systematically evaluated these risks in emergency or high-pressure clinical scenarios, which represents a critical gap in the literature.

While the BC-SSI architecture may mitigate risks associated with centralized identity repositories, it also redistributes responsibility between users and institutions, requiring careful governance and usability design. This trade-off demonstrates that security mechanisms must be evaluated not only for technical robustness but also for their impact on patient safety and clinical workflow reliability.

### Platform Selection and Architectural Trade-Offs

The comparative evaluation of the blockchain platforms reveals significant architectural divergences. Hyperledger Indy, often combined with Aries, dominates identity-centric implementations because of its native support for DIDs and VCs. Permissioned platforms such as Hyperledger Fabric and Besu offer enterprise-grade governance and controlled participation models, aligning more naturally with regulated health care environments. Public Ethereum-based solutions provide programmability and a mature smart contract ecosystem but raise concerns about transaction transparency, scalability, and regulatory suitability. These choices are not merely technical preferences but reflect underlying governance assumptions, trust models, and regulatory constraints.

In addition, nearly one-third of the studies failed to clearly specify their blockchain infrastructure, thereby limiting reproducibility and technical assessment. This lack of architectural transparency constitutes a methodological weakness in the current literature and limits the ability to assess the suitability of proposed solutions for real-world health care deployment. This demonstrates that platform selection is a critical architectural decision that directly shapes privacy guarantees, interoperability potential, and regulatory alignment.

### Stakeholder Implications and Systemic Constraints

The potential of BC-SSI cannot be evaluated solely through a technical lens. Its feasibility depends on stakeholder alignment across patients, providers, regulators, and system developers.

For patients, SSI promises granular control over identity attributes and consent. However, user-facing challenges, including wallet management, credential recovery, cognitive load, and emergency override procedures, are rarely evaluated through formal usability studies. For example, in acute care scenarios, delayed identity recovery or inaccessible credentials could directly affect continuity of care. The limited exploration of recovery frameworks and emergency access models represents a critical gap between conceptual autonomy and practical safety.

For health care providers, integration with established clinical workflows remains largely unaddressed. Limited studies demonstrated integration with existing EHR or hospital information systems, or comprehensive alignment with standards such as Health Level Seven (HL7) and Fast Healthcare Interoperability Resources (FHIR). Without such integration, BC-SSI remains peripheral to routine care delivery, limiting its practical impact on health care operations.

For regulators and policymakers, the distinction between technical privacy preservation and demonstrable legal compliance is crucial. Although the BC-SSI architecture conceptually aligns with GDPR and HIPAA principles, few studies have evaluated audit mechanisms, credential revocation governance, issuer accreditation models, or the legal enforceability of VCs. Governance design, particularly schema ownership, trust registries, and cross-jurisdictional interoperability, remains underdeveloped. This demonstrates that governance is a primary barrier to adoption rather than a secondary design consideration.

Taken together, these findings indicate that BC-SSI maturity is constrained less by cryptographic capabilities than by governance, interoperability, and human-centered implementation challenges.

### Conceptual BC-SSI Health Care Architecture

This section presents a novel contribution of this review by moving beyond descriptive analysis toward an integrated architectural perspective. The synthesis of the reviewed studies indicates that BC-SSI implementations in health care can be understood as a multilayered architecture integrating identity infrastructure, blockchain platforms, privacy-preserving mechanisms, health care system interoperability, and governance structures. While many studies emphasize decentralized identity technologies and cryptographic mechanisms, these components are often addressed in isolation rather than as integrated systems.

**Figure 3:**
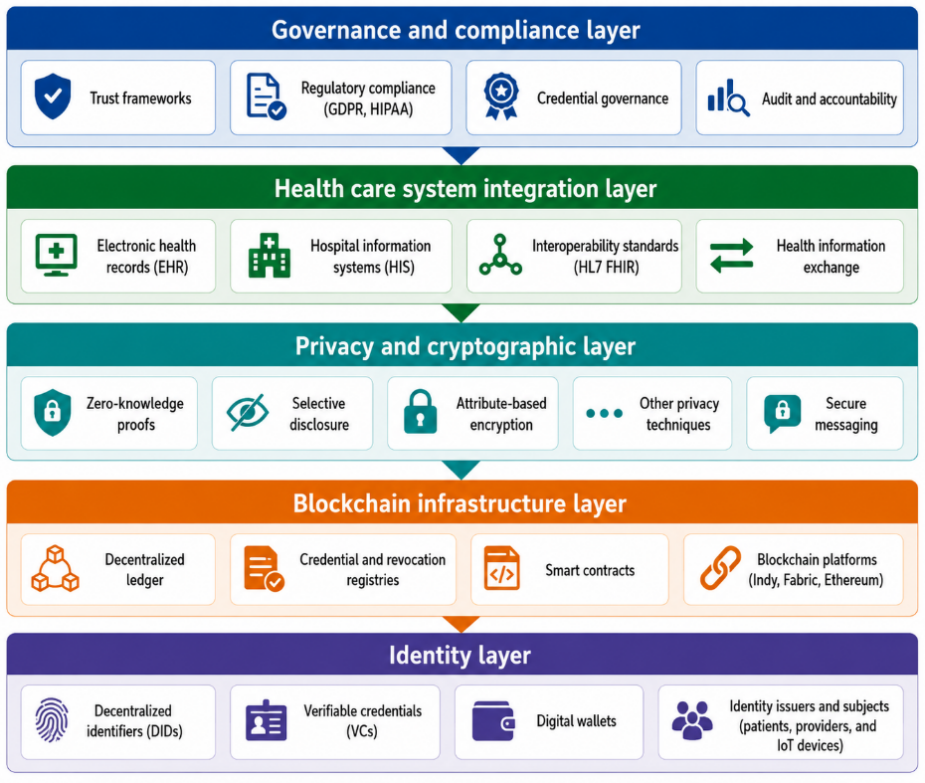
Conceptual multilayered blockchain-enabled self-sovereign identity (BC-SSI) health care architecture. FHIR: Fast Healthcare Interoperability Resources; GDPR: General Data Protection Regulation; HIPAA: Health Insurance Portability and Accountability Act; HL7: Health Level Seven; IoT: internet of things.

Based on the recurring elements identified across the literature, Figure 3 illustrates a conceptual BC-SSI health care architecture that synthesizes these patterns into a unified system-level perspective.

At the foundational level, the identity layer includes DIDs, VCs, and digital wallets that enable patients, providers, and health care devices to manage decentralized digital identities. These identities are supported by the blockchain infrastructure layer, which maintains credential schemas, revocation registries, and verification records using distributed-ledger platforms such as Hyperledger Indy, Fabric, and Ethereum-based systems.

A privacy and cryptographic layer enables secure verification through mechanisms such as selective disclosure, ZKPs, and cryptographic signatures, allowing health care attributes to be validated without exposing unnecessary personal data. These capabilities interact with the health care system integration layer, in which BC-SSI identity verification interfaces with EHR, hospital information systems, and health care interoperability standards such as HL7 and FHIR.

Finally, a governance and compliance layer provides the regulatory and organizational framework necessary for health care adoption. This layer encompasses credential governance policies, issuer accreditation, trust registries, and compliance with regulatory frameworks such as GDPR and HIPAA.

This layered perspective highlights that current research lacks a holistic architectural approach, limiting its ability to support end-to-end health care identity systems.

### From Prototype Innovation to Clinical Readiness

When interpreted through the conceptual architecture illustrated in Figure 3, the findings reveal a structural imbalance in current BC-SSI health care research, with a disproportionate focus on technical components rather than system-level integration. Most reviewed studies concentrate on the lower layers of the architecture, particularly the identity and blockchain infrastructure layers, in which DIDs, VCs, and distributed ledger technologies have been widely implemented and experimentally validated.

In contrast, the upper layers of architecture remain comparatively underdeveloped. Integration with clinical infrastructures, including EHR systems and interoperability standards such as HL7 and FHIR, is rarely demonstrated in existing prototypes. Similarly, governance mechanisms, including credential trust registries, issuer accreditation models, revocation governance, and regulatory alignment with frameworks such as GDPR and HIPAA, are frequently discussed conceptually but seldom implemented or evaluated in operational environments. This demonstrates that current research does not adequately address the requirements for real-world health care deployment.

These observations suggest that the maturity of BC-SSI in health care is constrained less by cryptographic capability than by challenges related to governance design, interoperability alignment, and human-centered implementation. While technical innovation in decentralized identity and credential architectures is advancing rapidly, real-world health care deployment remains limited due to insufficient evaluation of cross-institution workflows, credential lifecycle management, emergency access mechanisms, and usability of patient-facing identity tools.

Rather than evaluating BC-SSI systems solely in terms of functional capabilities, this review demonstrates that clinical readiness depends on system-level integration, governance alignment, and real-world validation. This indicates that the primary barrier to BC-SSI adoption is not technological feasibility but the absence of coordinated validation across technical, organizational, and regulatory dimensions.

### Synthesis Across Research Questions

Taken together, the findings of this review provide a coherent response to the RQs. RQ1 and RQ2 are addressed by demonstrating that BC-SSI applications are concentrated in identity-centric use cases and remain largely at the conceptual or prototype stage, with limited progression toward real-world deployment. RQ3 is addressed by identifying the dominant study aims, particularly privacy preservation, secure data exchange, and patient-centric identity control. RQ4 is addressed by revealing that the challenges targeted by these studies, primarily privacy, security, and interoperability, are approached predominantly through technical mechanisms, with limited consideration of operational, governance, and integration constraints.

The discussion further extends these findings by demonstrating that the key limitation of current BC-SSI research is not a lack of technical capability but the absence of system-level validation, governance alignment, and integration with health care infrastructures. This synthesis reinforces the central gap identified in this study and highlights the conditions necessary for advancing BC-SSI toward clinical readiness.

### Limitations

This scoping review has several limitations. First, it included only peer-reviewed English-language publications from 2015 to 2025. Relevant gray literature, industrial white papers, pilot deployments, or non-English studies may therefore not be represented. Given the rapidly evolving nature of blockchain and SSI ecosystems, some practical implementations may exist outside academic reporting.

Second, consistent with scoping review methodology, this study did not perform a formal quality appraisal or risk-of-bias assessment of included studies. The objective was to map the breadth and characteristics of BC-SSI research rather than evaluate intervention effectiveness. As a result, findings should be interpreted as a structured synthesis of existing proposals and implementations rather than a quantitative assessment of performance or security robustness.

Third, heterogeneity in terminology across BC-SSI research may have influenced retrieval. Although comprehensive Boolean search strategies and snowballing were used, emerging terminology or alternative phrasing may have led to the exclusion of the relevant studies.

Despite these limitations, adherence to the Arksey and O’Malley framework and PRISMA-ScR guidelines enhances transparency and reproducibility, providing a systematic overview of current BC-SSI health care research. The conceptual architecture presented in this study should therefore be interpreted as a synthesis derived from the reviewed literature rather than as an empirically validated system design.

### Implications and Future Research Directions

The findings of this review have important implications for both research and practice. First, it demonstrates that advancing BC-SSI in health care requires a shift from technology-centric development toward system-level design and validation. While current studies successfully demonstrate cryptographic feasibility, their limited integration with health care workflows, governance frameworks, and regulatory requirements constrains their practical applicability. This suggests that future BC-SSI research must adopt a sociotechnical perspective that considers not only technical performance but also usability, interoperability, and organizational alignment.

Second, the proposed conceptual architecture highlights the need for coordinated development across multiple layers, including identity infrastructure, blockchain platforms, privacy-preserving mechanisms, health care system integration, and governance frameworks. This layered perspective provides a structured foundation for designing and evaluating BC-SSI systems in health care and may serve as a reference model for future implementations.

Future research should prioritize multi-institutional pilot deployments that evaluate BC-SSI systems under realistic health care conditions. This includes assessing interoperability across health care providers, integration with EHR systems, and alignment with established standards such as HL7 and FHIR. Longitudinal evaluations are particularly important for assessing system scalability, performance, and usability over time.

Governance development represents a critical research frontier. Formal trust frameworks, credential schema governance models, revocation mechanisms, and issuer accreditation processes must be systematically designed and validated. In addition, regulatory mapping studies are required to explicitly align decentralized identity operations with frameworks such as GDPR and HIPAA, moving beyond conceptual compliance toward demonstrable accountability.

Human-centered evaluation is equally essential. Future studies should examine usability challenges related to identity wallets, credential recovery, and emergency access mechanisms to ensure that patient autonomy does not compromise safety or continuity of care. Security assessments should also consider real-world threat scenarios, including wallet compromise, key loss, correlation attacks, and cross-institutional vulnerabilities.

Collectively, these directions highlight that the future of BC-SSI in health care depends not only on advancing cryptographic techniques but also on achieving integration, governance maturity, and clinical validation.

### Conclusion

This scoping review systematically analyzed 37 peer-reviewed studies investigating BC-SSI applications in health care. The findings indicate that, while research activity has increased significantly in recent years, the field remains characterized by conceptual and prototype-driven development, with limited evidence of real-world deployment or clinical validation. The review demonstrates that BC-SSI research is primarily focused on identity-centric use cases, including credential verification, authentication, and privacy-preserving data exchange. These applications directly address longstanding challenges in health care identity management, particularly those related to privacy, security, and fragmented trust models. However, a clear gap persists between technical capability and operational readiness.

By synthesizing findings across studies, this review shows that the primary limitation of current BC-SSI research is not technological feasibility but the lack of integration with health care systems, governance frameworks, and regulatory requirements. This study therefore advances current literature by bridging the gap between fragmented technical proposals and system-level understanding required for health care implementation. This gap is further highlighted through the proposed conceptual multilayered architecture, which reveals a structural imbalance between well-developed technical components and underdeveloped system-level elements such as interoperability, governance, and clinical integration.

In addressing the RQs, this study provides a comprehensive mapping of BC-SSI applications (RQ1), identifies their developmental maturity (RQ2), analyzes their primary aims (RQ3), and examines the challenges they seek to address (RQ4). Beyond descriptive synthesis, this review contributes an analytical perspective by reframing BC-SSI maturity in terms of translational readiness, emphasizing the importance of system-level validation and real-world applicability.

BC-SSI technologies hold significant potential to enhance patient-centric identity management and secure data exchange in health care. However, their successful adoption will depend on coordinated progress across technical, organizational, and regulatory domains. Future research must therefore move beyond isolated technical innovation toward integrated, governance-aware, and clinically validated identity systems. Only through such efforts can BC-SSI transition from conceptual promise to a reliable foundation for health care identity infrastructures.

## Appendix

Multimedia Appendix 1 PRISMA-ScR checklist.

Multimedia Appendix 2 Full search strings.

## Abbreviations

BC-SSI: blockchain-enabled SSI
DID: decentralized identifier
EHR: electronic health record
FHIR: Fast Healthcare Interoperability Resources
GDPR: General Data Protection Regulation
HIPAA: Health Insurance Portability and Accountability Act
HL7: Health Level Seven
mHealth: mobile health
PRISMA-ScR: Preferred Reporting Items for Systematic Reviews and Meta-Analyses Extension for Scoping Reviews
RQ: research question
SSI: self-sovereign identity
VC: verifiable credential
ZKP: zero-knowledge proof

## Data Availability

All data analyzed in this study were derived from published peer-reviewed literature. The extracted data supporting the findings of this study are available from the corresponding author on reasonable request.
