# Supplementary material for: Blockchain-Enabled Self-Sovereign Identity Applications in Health Care: Scoping Review
Source: J Med Internet Res. 2026 May 26;28:e89574. doi: 10.2196/89574 (PMC13250497; doi:10.2196/89574)
Supplement: Multimedia Appendix 2 [file jmir_v28i1e89574_app2.docx]

## Multimedia Appendix 1

#### Scopus

TITLE-ABS-KEY (health* OR ehealth* OR e-health* OR "e health*" OR medic* OR clinic* OR patient* OR hospital*) AND TITLE-ABS-KEY ("self-sovereign identity" AND blockchain)

#### IEEE Xplore

(health* OR ehealth* OR e-health* OR medical OR medicine OR clinic* OR patient OR patients OR hospital OR hospitals) AND ("self-sovereign identity" AND blockchain)

#### Google Scholar

((health* OR ehealth* OR e-health* OR "e-health*" OR medic* OR clinic* OR patient* OR hospital*) AND ("Self-sovereign identity" OR blockchain))

#### Web of of Science

TS=((health* OR ehealth* OR e-health* OR "e health*" OR medic* OR clinic* OR patient* OR hospital*) AND ("self-sovereign identity" AND blockchain))

#### Embase And Medline

(health* OR ehealth* OR ’e health*’ OR ’e-health*’ OR medic* OR clinic* OR patient* OR hospital*) AND (’self sovereign identity’ AND blockchain)
